# Shiga toxin-producing *Escherichia coli* infection as a precipitating factor for atypical hemolytic-uremic syndrome

**DOI:** 10.1007/s00467-024-06480-9

**Published:** 2024-09-30

**Authors:** Gabriele Mortari, Carolina Bigatti, Giulia Proietti Gaffi, Barbara Lionetti, Andrea Angeletti, Simona Matarese, Enrico Eugenio Verrina, Gianluca Caridi, Francesca Lugani, Valerio Gaetano Vellone, Decimo Silvio Chiarenza, Edoardo La Porta

**Affiliations:** 1 UOC of Nephrology, Dialysis and Transplantation, IRCCS Istituto Giannina Gaslini, Genoa, Italy; 2 Department of Medical and Surgical Specialties, Radiological Sciences and Public Health, University of Brescia, Brescia, Italy; 3 Department of Neurosciences, Rehabilitation, Ophthalmology, Genetics, Maternal and Child Health, University of Genoa, Genoa, Italy; 4 UOC of Pathology, IRCCS Istituto Giannina Gaslini, Genoa, Italy; 5 UOC of Nephrology, Dialysis and Transplantation, ASST Spedali Civili, Brescia, Italy

**Keywords:** Atypical HUS, STEC-HUS, Eculizumab, Anti-complement therapy, Anti-CFH antibody, CFHR

## Abstract

**Background:**

Hemolytic uremic syndrome (HUS) is a thrombotic microangiopathy characterized by intravascular hemolysis. It can be classified as either typical, primarily caused by Shiga toxin-producing *Escherichia coli* (STEC) infection, or as atypical HUS (aHUS), which results from uncontrolled complement activation.

**Methods:**

We report the case of a 9-year-old boy with aHUS due to compound heterozygous complement factor H-related genes (CFHR) *1/3* and *CFHR1*–*CFHR4* deletions, leading to the development of anti-complement factor H (CFH) autoantibodies. The patient presented nephrological and neurological thrombotic microangiopathy with STEC positivity. Additionally, we provide an extensive literature review of aHUS cases initially classified as typical.

**Results:**

A total of 11 patients were included, 73% of whom were pediatric. Kidney replacement therapy was required in 73% of patients. The recurrence rate was 55%. All cases were found positive for pathological variants of the complement system genes. The most commonly implicated gene was *CFH*, while the *CFHR* genes were involved in 36% of cases, although none exhibited anti-CFH autoantibodies. Anti-complement therapy was administered in 54% of cases, and none of the patients who received it early progressed to kidney failure.

**Conclusions:**

STEC infection does not exclude aHUS diagnosis, and early use of anti-complement therapy might be reasonable in life-threatening conditions. Genetic testing can be helpful in patients with atypical presentations and can confirm the necessity of prolonged anti-complement therapy.

**Graphical Abstract:**

**Figure:**
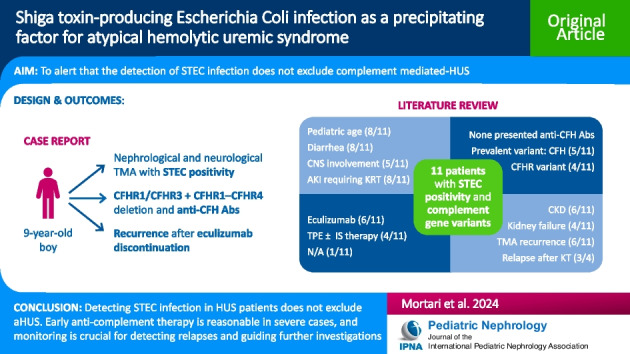
A higher resolution version of the Graphical abstract is available as Supplementary information

**Supplementary Information:**

The online version contains supplementary material available at 10.1007/s00467-024-06480-9.

## Introduction

Thrombotic microangiopathy (TMA) is a rare condition affecting both children and adults, characterized by hemolytic microangiopathic nonimmune anemia, thrombocytopenia, and acute kidney injury (AKI). TMA is classified as hemolytic uremic syndrome (HUS), thrombotic thrombocytopenic purpura (TTP), and secondary TMA. HUS is categorized as either typical or atypical, based on pathogenesis. Typical HUS, responsible for 80–90% of cases [1, 2], is caused by a Shiga toxin-producing (Stx) enteropathogenic bacterial infection (most commonly the Shiga toxin-producing *Escherichia coli* (STEC)) and is termed STEC-HUS [3]. Typical HUS mostly affects children and usually presents with bloody diarrhea, with AKI requiring kidney replacement therapy (KRT) in around 50% of cases [4], which may evolve into chronic kidney disease (CKD) in 20–30% of AKI [5].

Although aHUS occurs less frequently than STEC-HUS [4], it typically presents with more severe symptoms and can potentially progress with a more aggressive disease course [3]. aHUS results from a congenital or acquired alteration of the complement alternative pathway, necessitating chronic treatment to prevent disease recurrence. Additionally, certain rare forms of aHUS are caused by variants in genes unrelated to the complement system, such as diacylglycerol kinase epsilon (*DGKE*) and thrombomodulin (*THBD*).

In the last two decades, the development of the monoclonal antibody eculizumab, which targets complement protein C5 and prevents cleavage to C5a and C5b, has reduced the mortality rate of this condition [6]. Despite the absence of established clinical guidelines, the administration of eculizumab may be considered appropriate in STEC-HUS cases as a rescue therapy, particularly in life-threatening situations, especially those with severe neurological complications [7–9].

Distinguishing between STEC-HUS and aHUS poses a clinical challenge due to the absence of specific distinguishing features. The diagnostic tests for these conditions are often time-consuming and costly, complicating therapeutic decision-making for clinicians. aHUS is a diagnosis per exclusion, and STEC-HUS is the first diagnosis to rule out. Nevertheless, in very rare cases, a STEC infection and a genetically based or acquired alteration in the complement pathway may coexist. According to the “two-hit” mechanism model, a STEC infection can trigger a complement-mediated form of HUS in patients with a predisposing genetic background. Additionally, STEC-HUS could be more severe in patients with variants in complement genes. The identification of STEC does not rule out the presence of a complement pathway alteration, and this potential overlap should be considered in specific clinical situations.

We present the case report of a young boy presenting with AKI and neurological involvement due to TMA and resulting positive for STEC O157. Further investigations revealed a high serum level of anti-complement factor H (CFH) autoantibodies and pathological variants of complement factor H-related (*CFHR*) genes. We also present a systematic literature review of similar cases. Moreover, we critically review the role of complement in STEC-HUS, indications for genetic analysis, and the use of anti-complement therapy.

## Case report

**Fig. 1:**
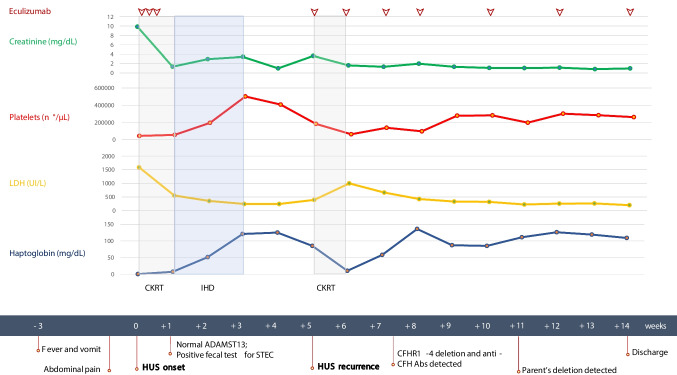
Timeline of the most important clinical events. On the upper graph, trends in biochemical parameters are reported. Below, the most important clinical and laboratory findings are reported. *CKRT*, continuous kidney replacement therapy; *IHD*, intermittent hemodialysis; *LDH*, lactate dehydrogenase; *HUS*, hemolytic uremic syndrome; *ADAMST13*, a disintegrin and metalloproteinase with thrombospondin type 1 motif member 13; *STEC*, Shiga toxin-producing *Escherichia coli*; *CFH*, complement factor H; *CFHR*, complement factor H-related genes; *Abs*, antibodies

A 9-year-old boy presented fever and vomiting, without diarrhea, which resolved spontaneously in a week. After 3 weeks, he was hospitalized at the Giannina Gaslini Children’s Hospital, Genoa, Italy, due to anuria and epileptic seizures. He presented severe hypertension (140/90 mmHg), and serum tests revealed AKI stage KDIGO 3 (creatinine: 9.86 mg/dL, urea: 355 mg/dL), microangiopathic anemia (hemoglobin: 7.4 g/dL, lactate dehydrogenase: 1585 IU/L, haptoglobin: < 2 g/dL), thrombocytopenia (platelets: 48,000/mm^3^), low C3 level (59 mg/dL), and cardiac involvement (NT-terminal pro B-type natriuretic peptide: 14,832 pg/mL, and septal hypokinesia) (Fig. 1). Given the severe neurological and kidney involvement, therapy with the monoclonal humanized anti-C5 antibody eculizumab was initiated, supplemented by supportive therapies such as continuous kidney replacement therapy (CKRT), plasma and blood transfusions, and intravenous antihypertensive drugs. According to the common clinical practice and the manufacturer’s suggestions, eculizumab was administered at a dose of 600 mg, followed by two additional doses of 300 mg following daily plasma infusions. After three doses of eculizumab, treatment was discontinued due to clinical improvement. Plasma and blood transfusions were stopped, and CKRT was shifted to intermittent hemodialysis until the recovery of kidney function at day 24.

Serum metalloproteinase ADAMTS13 (a disintegrin and metalloproteinase with a thrombospondin type 1 motif, member 13) activity was within normal range. Polymerase chain reaction analysis of a stool sample collected upon admission detected virulence genes encoding Shiga toxins 1 and 2 (Stx1, Stx2) and identified STEC O157. Consequently, a presumptive diagnosis of STEC-HUS was made.

During the fifth week of hospitalization, the patient presented nausea, abdominal pain, vomiting, and severe hypertension. Laboratory tests indicated AKI recurrence (creatinine: 3.86 mg/dL), and proteinuria onset, initially without signs of hemolysis. Histological investigation of the kidney showed renal TMA, characterized by intravascular thrombosis, vascular wall thickening, and cellular crescents. The head magnetic resonance imaging revealed severe posterior reversible encephalopathy syndrome (PRES) with associated cortical cytotoxic edema. The patient needed readministration of CKRT and support therapies. Hemolytic anemia and thrombocytopenia manifested in the following days, prompting the resumption of eculizumab infusions due to suspicion of aHUS.

Genetic tests performed via next-generation sequencing (NGS) panel and multiplex ligation-dependent probe amplification (MLPA) revealed compound heterozygous *CFHR1*/*CFHR3* and *CFHR1*–*CFHR4* gene deletions. Furthermore, an enzyme-linked immunosorbent assay (ELISA) test detected high serum antibody titers against CFH (222 UI/mL; n.v.: < 5.3 IU/mL). Parental genetic analysis showed heterozygous deletion in the *CFHR1–CFHR4* genes (maternal) and heterozygous deletion in the *CFHR3–CFHR1* gene (paternal). Weekly administration of eculizumab was effected for 4 weeks, then every 2 weeks, leading to gradual improvement of clinical conditions. However, kidney function did not completely recover and progressed to CKD stage IIIa, with an estimated glomerular filtration rate (eGFR) of 57 mL/min/1.73 m^2^ 2 months from onset. Subsequently, the patient was switched to the long-acting C5 blocker ravulizumab.

## Methods

### Study guidelines

This review was conducted in conformity with the Preferred Reporting Items for Systematic reviews and Meta-Analyses statement (PRISMA). We searched manuscripts reporting cases with initial diagnosis of STEC-HUS followed by a subsequent diagnosis of aHUS.

### Search strategy and study eligibility

The literature search was carried out systematically (until December 2023) using the PubMed and Scopus databases using the following keywords: (“STEC” OR “typical HUS” OR “Shiga toxin” OR “HUS” OR “Escherichia coli”) AND (“complement gene mutation” OR “CFH antibodies” OR “anti-complement therapy” OR “eculizumab”). A manual search of relevant studies was also performed to integrate the systematic review. Duplicates were excluded, and two of the authors (M. G. and B. C.) independently reviewed titles and abstracts. Those articles considered pertinent were selected for full-text examination. Full-text examination was performed independently by two of the authors (P. G. G. and L. B.).

### Inclusion and exclusion criteria

Inclusion criteria included articles describing a case report, case series, and systematic review regarding STEC-HUS associated with pathogenic or likely pathogenic variants of complement genes, according to a database of complement gene variants (complement-db.org), or anti-CFH autoantibodies. Papers in languages other than English were not considered.

## Results

**Fig. 2:**
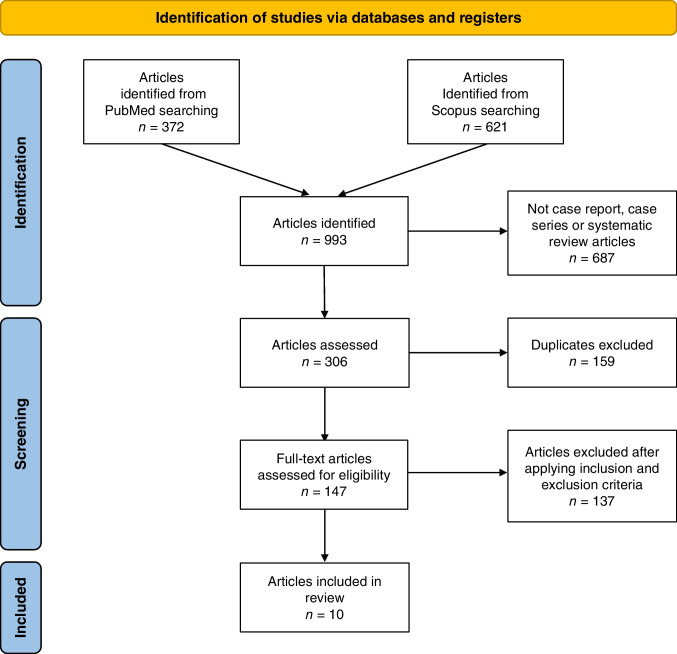
Flow chart of the methodology used to screen the articles. Ten articles published between 2008 and 2023 were included in the review

The first literature search revealed 993 potentially relevant articles. We then filtered for systematic review, case report, and case series (*n* = 306), and after duplicate exclusion, full-manuscript analysis was performed on 147 papers (Fig. 2).

After applying inclusion and exclusion criteria, a total of 10 articles were considered, for a total of 11 patients. Considered articles allowed the comparison of clinical manifestations at the onset and during acute phases, outcome, genetic characterizations, treatment, and recurrence rate analysis (Table 1).

**Table 1:** Overview of case reports included in the literature review

Source	Number of cases	Clinical manifestation at onset	Age at disease onset	Detection of STEC	Neurologic involvement	Dialysis	Treatment	Kidney biopsy	Outcome	Recurrence	Serologic status	Genetic findings
Alberti et al. [10], 2013	2	Pt (1): Watery diarrhea, profuse vomiting	Pt (1): 26 yo, female	Pt (1): Positivity for anti-Stx and anti-*E. coli* O157:H7 lipopolysaccharide antibodies in serum	Pt (1): Seizures and hypertensive encephalopathy	Pt (1): Hemodialysis	TPE	Pt (1): Classic histological features of thrombotic microangiopathy, diffuse and intense reactivity for C3 and C9	Pt (1): Bilateral nephrectomy	HUS relapsed after allogenic cadaveric kidney transplantation	Pt (1): Low C3 levels	Pt (1): *CFI*:c.1234G > A heterozygous
		Pt (2): Watery diarrhea, profuse vomiting	Pt (2): 16 yo, female	Pt (2): *E. coli* O157:H7 producing Stx1 and Stx2 in stool cultures	Pt (2): Not reported	Pt (2): Not reported	Pt (2): Not reported, only supportive therapy	Pt (2): Not reported	Pt (2): Kidney failure	HUS relapsed after living-donor kidney transplantation from her mother	Pt (2): normal C3, C4, CFH	Pt (2): *CD46*(MCP):c.286 + 2 T > G heterozygous
Galvez et al. [11], 2023	1	Somnolent and hypoactive, abundant diarrhea with fresh blood	4 yo, female	Positive stool culture and film array for entero-hemorrhagic *E. coli* O157:H7	Tonic–clonic seizure, need for mechanical ventilation, multiple cerebral ischemia associated with extensive cortical necrosis, and leukoencephalopathy	Peritoneal dialysis	Off-label eculizumab, first dose of 500 mg/m^2^, second dose 870 mg/m^2^ 1 week after the first	Not performed	GI involvement with massive digestive bleeding requiring colectomy with resection of the ascending, transverse, and descending colon No mention of CKD development	No	Low C3 serum levels	*CFH*:c.3572C > T(;)c.3590 T > C heterozygous
Caillaud et al. [12], 2015	1	Vomiting, non-bloody diarrhea, weight loss	18 months, male	Positive stool culture for *E. coli* and Stx2	None	Not required	Off-label eculizumab (two doses of 600 mg and 300 mg on consecutive days)	Not performed	Recovered completely	No	Low C3 levels, negative anti-CFH antibodies	*CFH*:c.2103G > A heterozygous
Celakil et al. [13], 2020	1	Bloody diarrhea	6 yo, male	Positive stool culture for *E. coli* O157: H7 and Stx1 and Stx2 strongly positive on PCR	None	Peritoneal dialysis	Two 600-mg doses of eculizumab Chronic eculizumab treatment after recurrence	Not performed	CKD	Relapse of HUS on day 10 after eculizumab discontinuation	Low C3 levels	*CFH*:c.2808G > T homozygous + CFB:c.94C > T heterozygous
Schwarz et al. [14], 2020	1	Intractable vomit, diarrhea, hypertension, skin purpura	34 yo, male	Stool culture positive for *E. coli* O128 and Stx2 and hemolysin via PCR	None	Not required	TPE Eculizumab after recurrence	Glomerular thrombotic microangiopathy with reduplication of basement membranes and prominent endothelial swelling	CKD	One month after onset recurrence of HUS Second recurrence after eculizumab discontinuation after 9 months of therapy	Normal levels of C3, C4, and CH50, negative anti-CFH antibodies	*CFH*-*CFHR1* heterozygous
Fang et al. [15], 2008	1	Hemorrhagic colitis, seizures, and anuria	4 yo, female	Positive stool culture for *E. coli* O55	Coma and respiratory insufficiency requiring mechanical ventilation	Hemodialysis	TPE	Not performed	Deceased due to multi-organ failure	No	Low C3, borderline low C4	*CD46*(MCP):c.998C > T heterozygous
Dowen et al. [16], 2017	1	4-day history of vomiting without diarrhea, AKI	16 yo, female	IgM antibodies to *E. coli* O157 and positive LPS	Seizures, attributed to hypertensive encephalopathy, 10 months after disease onset Recurrence of PRES post-transplant	Hemodialysis	TPE Eculizumab after recurrence post-transplantation	Features of TMA, segmental C3 deposition in the glomeruli	Kidne﻿y failure and deceased donor kidney transplantation	Recurrence of TMA after transplantation	Low levels of C3 and C4, normal CFH and CFI	*C3*:c.4855A > C heterozygous
Milan Manani et al. [17], 2017	1	Not described	2 yo, male	Not reported	None	Peritoneal dialysis and then hemodialysis	Not reported	Not reported	Progressed to kidney failure Two deceased donor kidney transplants, complicated by acute rejection and graft loss A third kidney transplant was performed with good results	No Histological examination of the grafts did not show signs of TMA	Not reported	*CFHR3*/*CFHR1* heterozygous deletion
McCoy et al. [18], 2014	1	Non-bloody diarrhea and abdominal pain, vomit, hypertension, confusion, and lethargy	10 yo, male	Positive ELISA test for *E. coli* producing Stx, negative stool culture	Lethargy and confusion	Hemodialysis	TPE	Presence of TMA	Recovered completely	No	Normal C3 and C4 level, negative anti-CFH antibodies	Heterozygous missense mutation in CFH (Gln950His) and a homozygous deletion in CFHR1 and CFHR3
Nalluru et al. [19], 2018	1	Fatigue, syncopal episode	43 yo, female	Positive stool Stx on PCR	Syncopal episode	Dialysis	Initially glucocorticoids and TPE and then eculizumab	Acute and subacute renal TMA involving glomeruli and vessels with secondary acute tubular injury	CKD	Recurrence after eculizumab discontinuation after 9 months of therapy	Low C3 levels, slightly decreased CFH, negative anti-CFH antibodies	Compound heterozygous deletion *CFHR3–CFHR1* ( +) *CFHR1–CFHR4*

*STEC*, Shiga toxin-producing *Escherichia coli*; *Pt*, patient; *yo*, years old; *Stx*, Shiga toxin; *TPE*, therapeutic plasma exchange; *HUS*, hemolytic uremic syndrome; *CFI*, complement factor I; *CKD*, chronic kidney disease; *CFH*, complement factor H; *MCP*, membrane cofactor protein; *CFB*, complement factor B; *PCR*, polymerase chain reaction; *CFHR*, complement factor H-related gene; *AKI*, acute kidney injury; *LPS*, lipopolysaccharide; *PRES*, posterior reversable encephalopathy syndrome; *TMA*, thrombotic microangiopathy; *ELISA*, enzyme-linked immunosorbent assay

### Clinical characteristics

Eight cases occurred during pediatric ages, while three patients were adults. Common symptoms at onset included diarrhea (8 out of 11 cases), with 3 cases (27%) exhibiting bloody diarrhea [11, 13, 15], and vomiting in 6 cases [10, 12, 14, 16, 18]. AKI requiring KRT was reported in 8 out of 11 cases [10, 11, 13, 15–19]. Neurological involvement was observed in five patients [10, 11, 15, 16, 18], predominantly characterized by seizures. PRES manifested in two out of five patients: one patient went into a coma [15], while the other presented ischemic lesions leading to subsequent necrosis and leukoencephalopathy [11].

### Genetic and serological findings

The predominant gene associated with variants was *CFH*, the regulatory protein of the alternative complement pathway. One patient had a homozygous *CFH* variant, associated with a heterozygous variant of complement factor B (*CFB*) [13], a serine protease that combines with C3b to generate the C3 or C5 convertase; two other patients had a heterozygous variant of *CFH*. One patient presented a heterozygous *CFH* missense variant [11, 12] associated with a homozygous deletion in *CFHR3*–*CFHR1* [18]. One patient was found to be a carrier of a heterozygous *CFHR1/CFH* hybrid gene [14]. Other genetic variants affected membrane cofactor protein (*MCP/CD46)*, a widely distributed C3b/C4b-binding cell surface glycoprotein which acts as an inhibitor of complement activation, in two patients [10, 15]. Another patient had a heterozygous variant in the C3 gene [16]. A variant of complement factor I (*CFI*), a serin protease which cleaves C3b and C4b in the presence of CFH and MCP, was found in one patient [10]. Interestingly, 64% of patients presented lower C3 levels, and circulating CFH antibodies were negative in all cases.

### Clinical outcomes

Six out of 11 patients developed CKD, while 4 progressed to kidney failure and received kidney transplantation [10, 16, 17]. One patient with a pediatric diagnosis of STEC-HUS and heterozygous deletion of *CFHR3–1* (but without circulating anti-CFH autoantibodies) received three kidney transplants. Graft loss was not attributed to TMA [17].

TMA recurred in 6 out of 11 patients: 3 cases with a native kidney and 3 cases after transplantation. Similar to our experience, two patients relapsed shortly after onset (10–30 days) [13, 14]. One patient had a relapse following eculizumab discontinuation after 9 months of therapy [19]. One patient had both early and late relapses [14]. The underlying genetic variations in these cases included homozygous *CFH* and heterozygous *CFB* variants [13], heterozygous *CFHR1/CFH* hybrid gene [14], and compound heterozygous deletion of *CFHR3–CFHR1* and *CFHR1–CFHR4* gene [19]. In patients with TMA recurrence after kidney transplantation, the following variants were observed: heterozygous missense *CFI* variant [10], heterozygous *C3* variant [16], and heterozygous *MCP* variant [10].

One patient developed digestive bleeding requiring colectomy [11], while one patient died due to multiorgan failure (MOF).

### Treatment strategies

As reported in Table 1, eculizumab was administered to 6 out of 11 patients, at different doses. Among these, four patients received early eculizumab treatment, resulting in the restoration of normal kidney function [11, 12] and in the development of CKD [13, 19] in 2 and 2 cases, respectively.

In the patient described by Galvez et al., eculizumab was administered due to severe neurological involvement. In the other three cases, anti-complement therapy was initiated based on suspicion of aHUS [11]. Caillaud et al. administered eculizumab to prevent dialysis, considering decreased C3 levels, persistent AKI, and ongoing hemolysis [12]. Similarly, Nalluru et al. administered eculizumab due to decreased C3 levels, slightly decreased CFH, and elevated CBb [19]. In the case described by Celakil et al. eculizumab was administered after a second episode of thrombocytopenia and hypocomplementemia on day 10, with poor kidney function improvement [13].

One patient received eculizumab after a TMA relapse 1 month after onset and subsequently developed CKD [14]. Another patient was treated with eculizumab following the TMA recurrence after kidney transplantation [16]. In patients not receiving eculizumab, four were treated with therapeutic plasma exchange (TPE) leading to different outcomes: two patients developed kidney failure and experienced relapse after kidney transplantation [10], one patient recovered kidney function [10], and one patient died due to MOF [15]. In one case, no treatment strategy was available [17] (Table 2).

**Table 2:** Different kidney outcomes in relation to eculizumab administration and complement variants

Patient	Eculizumab administration	Complement mutation	Kidney outcome
1 [10]	No	Heterozygous missense *CFI* mutation	Progression to KF Recurrence after KT
2 [10]	No	Heterozygous mutation in *MCP*	Progression to KF Recurrence after living KT
3 [11]	Yes	Two heterozygous mutations in *CFH*	Restore normal kidney function
4 [12]	Yes	Heterozygous mutation in *CFH*	Restore normal kidney function
5 [13]	Yes	Homozygous mutation in *CFH* and heterozygous mutation in *CFB*	CKD
6 [14]	Yes*	Heterozygous *CFHR1/CFH* hybrid gene	CKD
7 [15]	No	Heterozygous mutation in *MCP*	Deceased due to MOF
8 [16]	Yes †	Heterozygous rare genetic variant in *C3*	Progression to KF Recurrence after KT
9 [17]	N/A	Heterozygous deletion of *CFHR3–CFHR1*	Progression to KF No recurrence after KT
10 [18]	No	Heterozygous missense mutation in *CFH* and homozygous deletion in *CFHR1* and *CFHR3*	Restore normal kidney function
11 [19]	Yes	Heterozygous deletion of *CFHR3–1* and *CFHR1–4*	CKD

* Administered after recurrence. †Administered after recurrence following a kidney transplant. *CFI*, complement factor I; *KF*, kidney failure; *KT*, kidney transplantation; *MCP*, membrane cofactor protein; *CFH*, complement factor H; *CFB*, complement factor B; *CKD*, chronic kidney disease; *CFHR*, complement factor H-related gene; *MOF*, multi-organ failure; *N/A*, not available

## Discussion

The complement system is part of innate immunity and consists of numerous membrane-bound and circulating proteins that can be activated in different pathways, but which all converge to form the membrane attachment complex (MAC or C5b-9), which disrupts cell membrane integrity, leading to cell lysis. Several proteins participate in counteracting and modulating the persistent activation of the alternative pathway, thereby limiting damage to self-tissues. Pathogenic or likely pathogenic variants are normally identified in approximately 60% of aHUS cases.

Pathological variants of the *CFH* gene are the most prevalent in cases of aHUS [20], resulting in an excessive activation of the alternative pathway. The *CFH* gene and *CFHR1–CFHR5* are tandemly located on chromosome 1, exhibiting a significant level of sequence similarity. The presence of repetitive sequences promotes genomic rearrangements through nonallelic homologous recombination, leading to the identification of different hybrid genes resulting from recombination within the *CFH/CFHR* region in aHUS [21]. Homozygous deletions in *CFHR3–CFHR1* are commonly described in aHUS patients and are associated with the development of anti-CFH autoantibodies in 80% of cases [22]. We can speculate that *CFHR1* deficiency may increase the immunogenicity of CFH, determining the increase of anti-CFH autoantibodies [23], affecting the function of CFH [24]. Genetic analysis of our patient revealed a compound heterozygous deletion of *CFHR3–CFHR1* and *CFHR1–CFHR4*, resulting in the homozygous deletion of *CFHR1* and consequent generation of anti-CFH autoantibodies. STEC infection acted as a possible trigger for the clinical manifestation of aHUS, as previously described [10–19]. Identifying patients with aHUS is crucial due to the high risk of relapse and posttransplant recurrence. Anti-complement therapy has the potential to prevent recurrences and mitigate severe clinical consequences. This case prompts pertinent enquiry regarding the judicious timing of serological investigations and/or genetic testing in HUS. Under what circumstances is anti-complement therapy required? Is it effective in cases of STEC-HUS?

### Genetic test and complement alterations in HUS

The feasibility of genetic analysis for all HUS patients is constrained by availability and cost. In suspected cases of aHUS, prioritizing faster serological tests, including measurements of C3, C4, classical hemolytic 50% complement activity (CH50) levels, and screening for anti-CFH autoantibodies, would be advantageous. Identifying specific patient cohorts where genetic analysis is most appropriate becomes crucial, as it enables a strategic advantage in clinical outcomes.

Variants in complement genes usually involve *CFH*, *C3*, *CFI*, *CFB*, and *MCP*, as well as potential *CFHR1 − CFH* hybrid genes. To date, more than 500 variants in these five complement genes have been identified in patients with aHUS [25]. In these genes, rare variants with minor allele frequencies (MAFs) of < 1% and very rare MAFs of < 0.1% are present in 12% and 3.7% of healthy individuals, respectively [26, 27]. Variants with a MAF of < 0.1% might be considered relevant for the pathogenesis of aHUS or other complement-mediated disorders. Their relevance in other TMA associated diseases, including STEC-HUS, is still debated. In a US cohort/series, the frequency of rare complement variants is only moderately increased in patients with STEC-HUS compared with healthy individuals (16% vs. 12%) [28]. A comparison of the frequencies of rare and pathogenic variants in the five complement genes in healthy individuals and in patients with TMA suggests that only aHUS and pregnancy-associated HUS are associated with clear genetic susceptibility factors related to complement activation [29]. Therefore, genetic analysis would normally be limited to these conditions. However, diagnosing aHUS remains challenging as a genetic variant in the complement system alone is insufficient; a “second hit” is necessary. This second insult, precipitating the clinical manifestation of the disease, may arise from various sources, including bacterial infections (such as STEC infection), viral infections, drugs, pregnancy, and organ transplantation. Numerous conditions can autonomously contribute to the development of TMA, rendering the diagnostic process intricate, as underscored in our case report and comprehensive review.

Fremeaux-Bacchi et al. analyzed 108 children with post-diarrheal HUS, 75 Stx-positive and 33 Stx-negative cases [27]. They detected rare variants (< 1%) and very rare variants (< 0.1%) in *CFH*, *CFHR1–CFHR3*, *CFI*, *CFB*, *MCP*, *C3*, and *THBD* genes in 16% and 4% of STEC-positive patients. The frequency of rare variants was comparable to that found in 80 French controls (14%) and 503 healthy European subjects (12%). On the other hand, very rare variants were not identified in any of the French controls (0%) and in just 0.8% of European controls, indicating a fivefold increased risk for the development of HUS when associated with STEC-HUS. These findings suggested a genetic predisposition for complement activation in this context [27]. No significant differences were noted regarding neurological manifestations, AKI requiring KRT and/or the development of CKD. These findings indicate that the clinical impact of genetic variants on STEC-HUS is lower than that observed in aHUS. Nevertheless, a plausible scenario involves a multiple-hit model, suggesting that genetic factors, while less influential, may still contribute to the development of STEC-HUS.

As aHUS is prone to recurrence, meticulous patient follow-up is essential for accurate diagnosis and in mitigating potentially severe relapses. Due to the severe clinical manifestations and prognosis associated with aHUS, an early diagnosis becomes imperative to initiate timely and appropriate therapy. aHUS is definitely a diagnosis of exclusion, but as highlighted by the reported clinical case and literature review, the presence of a STEC infection should not automatically exclude a complement-mediated form of HUS. Indeed, the simultaneous presence of certain features such as the absence of bloody diarrhea, accentuated alternative complement pathway activation, and lack of recovery of kidney function should raise suspicion of additional pathogenetic mechanisms underlying HUS.

STEC-HUS patients require dialysis during the acute phase in approximately 50% of cases [4], with only 2–3% progressing to kidney failure and requiring kidney transplantation [30, 31].

In our small case series, dialysis was required in 72% of patients, of whom 36% progressed to kidney failure and requiring kidney transplantation, although 64% exhibited low C3 levels.

In the acute phase of STEC-HUS, low C3 levels are reported, particularly in severe cases [32]. However, a more pronounced activation of the alternative pathway is observed in aHUS compared to other forms of TMA [32], where hypocomplementemia occurs in up to 50% of cases [33]. Prohàszka et al. analyzed complement parameters in samples from 55 TMA patients and discovered that all patients with aHUS exhibited dysregulated alternative pathway activity and low C3 levels [34]. Compared to patients with TTP or STEC-HUS, patients with aHUS were more likely to demonstrate a dysregulation in the complement alternative pathway.

Sridharan et al. investigated the clinical utility of a complement serology panel (including CH50, alternative hemolytic 50% complement activity (AH50), C3, C4, CFB, CFH, C4d, Bb, and soluble MAC) in diagnosing aHUS within a cohort of 147 patients with TMA [35]. Their findings indicated that a decrease in both CFB and CH50 resulted in the best balance of sensitivity and specificity for diagnosing aHUS. Additionally, as noted by the authors, complement system abnormalities should be assessed in conjunction with clinical presentation. Despite the need for larger studies and the limited accessibility of these analyses, a comprehensive evaluation of the complement system shows potential to enhance physicians’ diagnostic capabilities in identifying atypical forms of HUS in cases with unclear etiopathogenesis. However, its current use is predominantly for research purposes rather than clinical practice, due to its limited availability in a few specialized centers and its time-consuming nature.

Kidney failure following STEC-HUS is uncommon, and genetic testing should be performed in patients who are being considered for kidney transplantation due to the high risk or recurrence tendency of complement-mediated HUS [36]. This becomes crucial in the context of living kidney transplantation from a related donor. For instance, the patient with an *MCP* variant described by Alberti et al. underwent transplantation from her mother, who shared the same *MCP* variant, resulting in the production of the same dysfunctional *MCP* protein in the graft and causing aHUS recurrence [10].

In summary, genetic screening and complement/serological investigations should be considered on a case-by-case basis for HUS patients with STEC infection who have an atypical presentation or disease course. This includes the absence of hematic diarrhea at onset, a family history of HUS or related parents, accentuated alternative pathway dysregulation, failure to recover kidney function, relapse, and posttransplant recurrence. Furthermore, in cases presenting as life-threatening, initiating anti-complement therapy at HUS onset should be considered, given the challenge of promptly establishing a definitive etiological diagnosis. Subsequent close monitoring of these patients is warranted to detect relapse, and if necessary, anti-complement therapy should be resumed and genetic analyses conducted. Moreover, population-specific cost-analysis investigation is recommended, since the above considerations may not always be readily applicable, especially in resource-limited countries.

### Anti-complement therapy in STEC-HUS

As widely reported, activation of the complement cascade is deeply involved in the pathogenesis of STEC-HUS [37–39]; however, the efficacy of anti-complement therapy is still debated.

Ahlenstiel-Grunow et al., in a case-series study, described 25 children with STEC-HUS, of whom 7 were treated with eculizumab [38]. The authors reported that high serum levels of C5b-9 significantly correlated with more severe clinical presentation in terms of arterial hypertension, edema, and lower platelet counts, but not with the need for prolonged KRT. Although patients in the eculizumab group exhibited a more severe HUS manifestation, eculizumab treatment did not appear to influence hematological outcome or the duration of KRT but showed a clear impact on the improvement of neurological symptoms in 11 out of 25 patients who presented with seizures and/or were in a stupor or coma. In a more recent multicentric study, Percheron et al. evaluated the use of eculizumab in 28 children with severe STEC-HUS, with neurological involvement [40]. Overall, 19 out of 28 had favorable neurological outcomes, 17 with prompt recovery following the first eculizumab dose.

In a systematic review of 21 STEC-HUS cases treated with eculizumab, the authors reported a rapid restoration of hematological (20 out of 21) and neurological (15 out of 21) parameters, while 19 out of 21 patients saw gradual improvement in kidney parameters [41]. Similarly positive outcomes were reported in more limited case series or case reports involving both pediatric [42, 43] and adult subjects [44].

Garnier et al. presented a randomized controlled multicenter trial addressing the efficacy and safety of eculizumab in 100 pediatric patients with STEC-HUS [45]. Authors showed that eculizumab was not associated with faster kidney improvement in the acute phase, but with better kidney outcomes, defined by high blood pressure and/or declined eGFR and/or proteinuria, at 1 year of follow-up. Furthermore, no disparity in hematological and extrarenal manifestations was observed between the two groups, although this study excluded patients with severe neurological presentations.

Most of the studies in the current literature indicate a cautious but positive clinical improvement in cases of severe STEC-HUS with neurological involvement. However, this evidence is based on mostly retrospective nonrandomized studies and case series; randomized controlled trials are required to determine the efficacy of eculizumab for such patients.

On the other hand, significant positive effect of eculizumab on medium- to long-term outcomes of 386 infection-associated HUS cases was observed in a systematic review by de Zwart et al., based on observational studies [46]. Nevertheless, the authors noted a critical risk of bias in most studies due to confounding factors. For instance, eculizumab administration was often delayed in sicker patients, potentially impacting its effectiveness; additionally, the co-administration of complement components with plasmapheresis might have attenuated eculizumab’s effect.

It is worth noting that genetic and/or complement abnormalities can be observed in STEC-HUS patients, whereas some cases of complement-mediated HUS are triggered by infection, making acute phase differentiation challenging. However, the treatment effect of eculizumab in STEC-HUS may be overestimated if the treatment group includes complement-mediated HUS patients. Nevertheless, the challenge in distinguishing between these patient groups during the acute phase may strengthen the case for the empirical treatment of STEC-HUS with eculizumab.

In conclusion, given the absence of other effective therapies, the potential benefits of eculizumab in patients with neurological involvement and the pathogenic role of complement dysregulation could justify its use in cases of severe HUS presentation at the onset where distinguishing between aHUS and STEC-HUS is challenging.

## Conclusion

Our experience and literature review suggest that the detection of STEC infection in a patient with HUS does not exclude a diagnosis of aHUS. In the presence of CNS involvement and/or life-threatening conditions, anti-complement therapy represents a reasonable approach at the onset of HUS, given the absence of other therapeutic resources.

Careful monitoring of such patients over time is crucial to promptly detect any disease relapses and initiate genetic investigations and prolonged complement-targeted therapy.

Investigations for autoantibodies and genetic factors could be helpful for a proper diagnosis in patients with atypical presentations, failure to recover kidney function, a family history of aHUS, or before kidney transplantation.

## Supplementary Information

Below is the link to the electronic supplementary material.

Graphical abstract (PPTX 93 KB)
