# Supplementary material for: Shiga toxin-producing Escherichia coli infection as a precipitating factor for atypical hemolytic-uremic syndrome
Source: Pediatr Nephrol. 2024 Sep 30;40(2):449–61. doi: 10.1007/s00467-024-06480-9 (PMC11666682; doi:10.1007/s00467-024-06480-9)
Supplement: Supplementary file 1 — Graphical abstract (PPTX 93 KB) [file 467_2024_6480_MOESM1_ESM.pptx]

## Slide 1
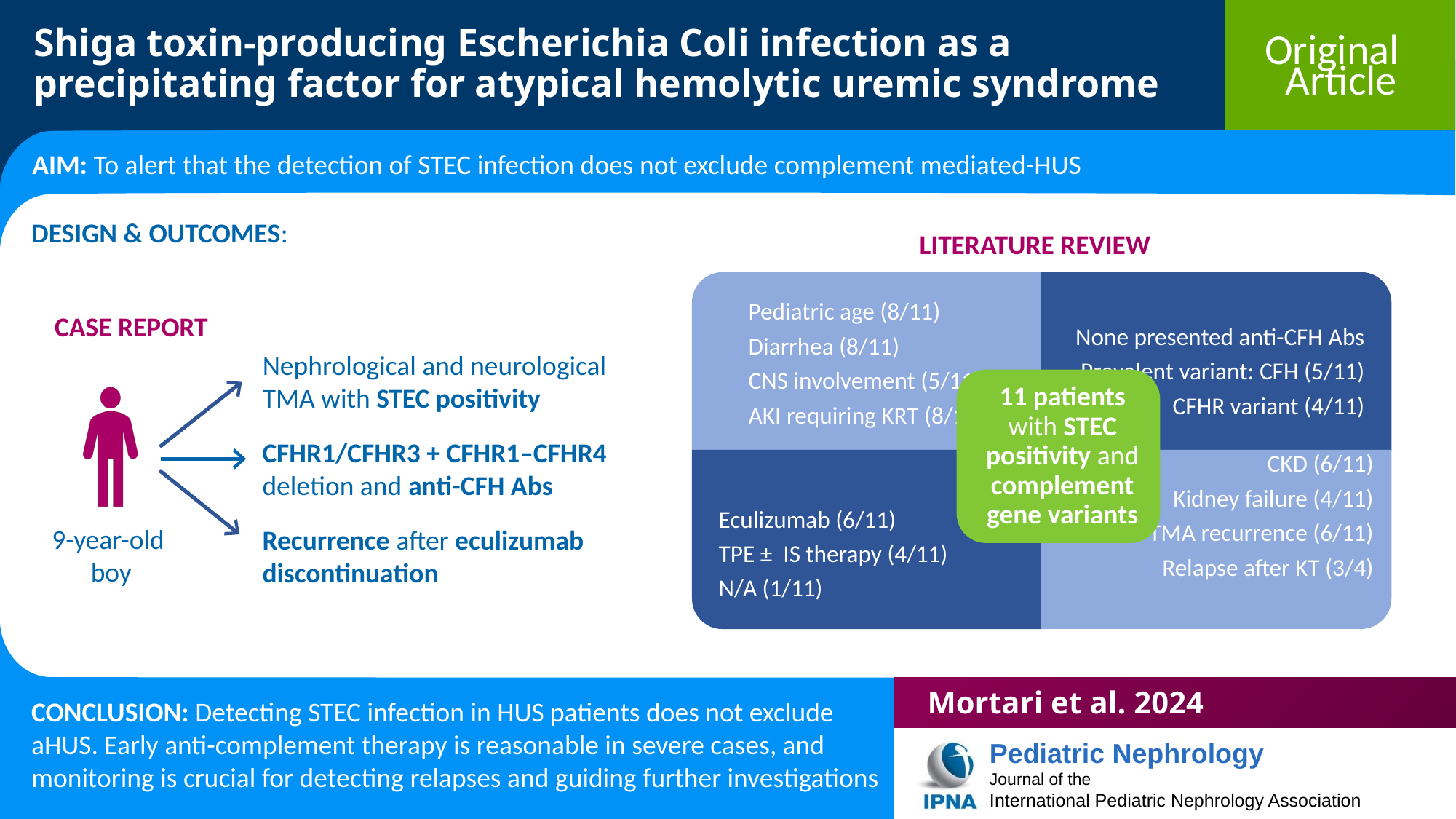

Shiga toxin-producing Escherichia Coli infection as a precipitating factor for atypical hemolytic uremic syndrome
AIM: To alert that the detection of STEC infection does not exclude complement mediated-HUS
DESIGN & OUTCOMES:
LITERATURE REVIEW
CASE REPORT
Nephrological and neurological TMA with STEC positivity
CFHR1/CFHR3 + CFHR1–CFHR4 deletion and anti-CFH Abs
9-year-old boy
Recurrence after eculizumab discontinuation
Mortari et al. 2024
CONCLUSION: Detecting STEC infection in HUS patients does not exclude aHUS. Early anti-complement therapy is reasonable in severe cases, and monitoring is crucial for detecting relapses and guiding further investigations
